# Comparison of the slow‐pull and aspiration methods of endobronchial ultrasound‐guided transbronchial needle aspiration for next‐generation sequencing‐compatible tissue collection in non‐small cell lung cancer

**DOI:** 10.1002/cam4.6561

**Published:** 2023-09-21

**Authors:** Yukihito Kajita, Shuhei Teranishi, Tomoe Sawazumi, Haruka Watanabe, Satoshi Nagaoka, Anna Tanaka, Yuichirou Suzukawa, Yuto Motobayashi, Tomofumi Hirose, Chihiro Maeda, Kenichi Seki, Ken Tashiro, Nobuaki Kobayashi, Masaki Yamamoto, Makoto Kudo, Yoshiaki Inayama, Takeshi Kaneko

**Affiliations:** ^1^ Respiratory Disease Center Yokohama City University Medical Center Yokohama Japan; ^2^ Division of Pathology Yokohama City University Medical Center Yokohama Japan; ^3^ Department of Pulmonology Yokohama City University Graduate School of Medicine Yokohama Japan

**Keywords:** clinical cancer research, next generation sequencing, non small cell lung cancer, oncogenes, target therapy

## Abstract

**Background:**

Personalized treatment for non‐small cell lung cancer (NSCLC) has advanced rapidly, and elucidating the genetic changes that trigger this disease is crucial for appropriate treatment selection. Both slow‐pull and aspiration methods of endobronchial ultrasound‐guided transbronchial needle aspiration (EBUS‐TBNA) are accepted methods for collecting samples suitable for next‐generation sequencing (NGS) to examine driver gene mutations and translocations in NSCLC. Here, we aimed to determine which of these two methods is superior for obtaining higher‐quality samples from patients with NSCLC.

**Methods:**

Seventy‐one patients diagnosed with NSCLC via EBUS‐TBNA using the slow‐pull or aspiration (20‐mL negative pressure) methods between July 2019 and September 2022 were included. A total of 203 tissue samples from the 71 patients were fixed in formalin, embedded in paraffin, and mounted on slides. The presence of tissue cores, degree of blood contamination, and number of tumor cells were compared between the groups. The success rate of NGS, using Oncomine Dx Target Test Multi‐CDx, was also compared between the groups.

**Results:**

The slow‐pull method was associated with a higher yield of tissue cores, lower degree of blood contamination, and higher number of tumor cells than the aspiration method. The success rate of the NGS was also significantly higher for the slow‐pull group (95%) than for the aspiration group (68%).

**Conclusion:**

Overall, these findings suggest that the slow‐pull method is a superior technique for EBUS‐TBNA to obtain high‐quality tissue samples for NGS. The slow‐pull method may contribute to the identification of driver gene mutations and translocations and facilitate personalized treatment of NSCLC.

## 1 INTRODUCTION

In recent years, personalized treatment for non‐small cell lung cancer (NSCLC) has advanced rapidly. Targeted drugs for advanced or recurrent NSCLC with driver gene mutations/translocations have improved prognoses, compared to cytotoxic anticancer drugs.^1^ In Japan, molecularly targeted drugs for epidermal growth factor receptor (*EGFR*) mutations,^2^ anaplastic lymphoma kinase (*ALK*) fusion gene,^3^ c‐ROS oncogene 1 (*ROS1*) fusion gene,^4^ v‐raf murine sarcoma viral oncogene homolog B1 (*BRAF*) gene V600E mutation,^5^ mesenchymal–epithelial transition factor gene exon 14 skipping mutation,^6^ rearranged during transfection (*RET*) fusion gene,^7^ Kirsten rat sarcoma viral oncogene homolog gene G12C mutation,^8^ and neurotrophic tyrosine receptor kinase fusion gene^9^ are covered by insurance. Previously, driver gene mutations/translocations were evaluated using individual companion diagnostic systems. Recently, next‐generation sequencing (NGS) has made it possible to search for them simultaneously. Furthermore, NGS is a powerful tool for diagnosing and treating NSCLC, as it can be used to monitor the response to treatment and detect resistance development.^10^ The use of NGS and the treatment of NSCLC are still evolving; however, NGS can potentially revolutionize the way that this cancer is managed.^11^ NGS requires smaller samples than sequential methods of individual genetic testing.^12^, ^13^, ^14^, ^15^, ^16^ However, the quality and quantity of nucleic acids in the specimen must meet specific standards, and a large number of tumor cells must be collected to ensure sufficient nucleic acid yield for analysis.^12^

Endobronchial ultrasound‐guided transbronchial needle aspiration (EBUS‐TBNA) is a safe and minimally invasive procedure that can be used to obtain tissue samples from bronchoscopically accessible hilar and mediastinal lesions. It is a standard method for obtaining tissue samples for lung cancer and has high diagnostic sensitivity and specificity.^17^ However, the success rate of NGS for EBUS‐TBNA varies between 46.8% and 98.8%.^14^, ^15^, ^16^, ^18^ This is because EBUS‐TBNA specimens are often contaminated with blood, which can lead to poor nucleic acid quality and adversely affect the success of NGS analysis.^13^, ^19^

In endoscopic ultrasound‐guided fine‐needle aspiration (EUS‐FNA) of pancreatic masses, many comparative studies have reported on the slow‐pull and aspiration methods.^20^, ^21^, ^22^ The slow‐pull method applies minimal aspiration force by slowly and continuously pulling a stylet without attaching a syringe; in contrast, the aspiration method applies negative pressure by attaching a syringe to the needle. Wang et al. conducted a meta‐analysis of previous studies and reported that the slow‐pull method allowed the collection of specimens with less blood contamination and had a better tissue core collection rate than the aspiration method.^23^ However, limited studies have compared the two techniques in EBUS‐TBNA, and it is unclear whether the slow‐pull method can be used to collect lung samples suitable for NGS with low blood contamination and high tumor cell counts. Therefore, in this study, we retrospectively compared the quality of specimens collected using the two techniques from patients diagnosed with NSCLC using EBUS‐TBNA.

## 2 PATIENTS AND METHODS

### 2.1 Study design and patients

This was a single‐center, retrospective study conducted at the Yokohama City University Medical Center, Japan. The study was conducted in accordance with the tenets of the Declaration of Helsinki and approved by the Ethics Committee of Yokohama City University (approval number: F221000009). Patients diagnosed with NSCLC using EBUS‐TBNA via the slow‐pull or aspiration method between July 2019 and September 2022 were included in this study. This study was retrospective in nature and thus did not require written consent from patients. Nevertheless, we published the study information on the hospital's website and offered patients the option to refuse participation.

The following patients were considered eligible for the study: (a) patients who underwent EBUS‐TBNA using the slow‐pull or aspiration methods between July 2019 and September 2022, (b) patients whose tissue samples were obtained using EBUS‐TBNA, and (c) patients diagnosed with NSCLC via tissue diagnosis. For patients who underwent both slow‐pull and aspiration procedures, specimens collected using both methods were combined for NGS analysis. Therefore, the success rates of NGS analysis for the two methods could not be compared and were excluded from this study. We collected patient background data including age, sex, smoking history, Eastern Cooperative Oncology Group performance status (ECOG PS), duration of the procedure, number of specimens collected, stage of NSCLC, pathological diagnosis, and programmed death ligand 1 (PD‐L1) tumor proportion score (TPS). Additionally, we collected data on the puncture site and the long diameter of the puncture site. Puncture sites were classified based on a lymph node map created by the International Association for the Study of Lung Cancer.^24^

### 2.2 EBUS‐TBNA procedure

The patients were placed under moderate‐to‐deep sedation with intravenous anesthesia (propofol and dexmedetomidine hydrochloride). A local anesthetic (2% lidocaine) was administered intratracheally during the examination. Blood pressure, pulse rate, and percutaneous oxygen saturation were monitored during the examination, and oxygen was administered to achieve percutaneous oxygen saturation ≥ 90%. EBUS‐TBNA was performed using an ultrasound bronchial fiber videoscope (BF‐UC260FW or BF‐UC290F; Olympus, Tokyo, Japan) and a 21‐gauge needle (NA‐U401SX‐4021; Olympus). After the delineation of lesions by EBUS, color Doppler ultrasound was used to evaluate the blood flow within and around the lesion to determine the optimal puncture site. A puncture needle was used to perforate the target mass, and a stylet was immediately pressed against the mass. The slow‐pull method was performed as follows: Approximately 20–40 strokes were performed while the stylet was slowly and continuously removed. Similarly, the aspiration method was performed as follows: The stylet was removed entirely, a 20‐mL negative pressure syringe was attached, and 20–40 strokes were performed. The steps were repeated several times until a sufficient number of specimens were obtained. The specimen in the puncture needle was pushed out with an air‐filled syringe and immediately placed in 10% neutral buffered formalin solution (Muto Pure Chemicals Co., Ltd.). All procedures were performed under the supervision of a respiratory physician with at least 8 years of experience in bronchoscopy. Owing to staffing issues in the pathology department, rapid on‐site evaluation was not performed in all cases.

### 2.3 Evaluation of specimen pathology

**FIGURE 1:**
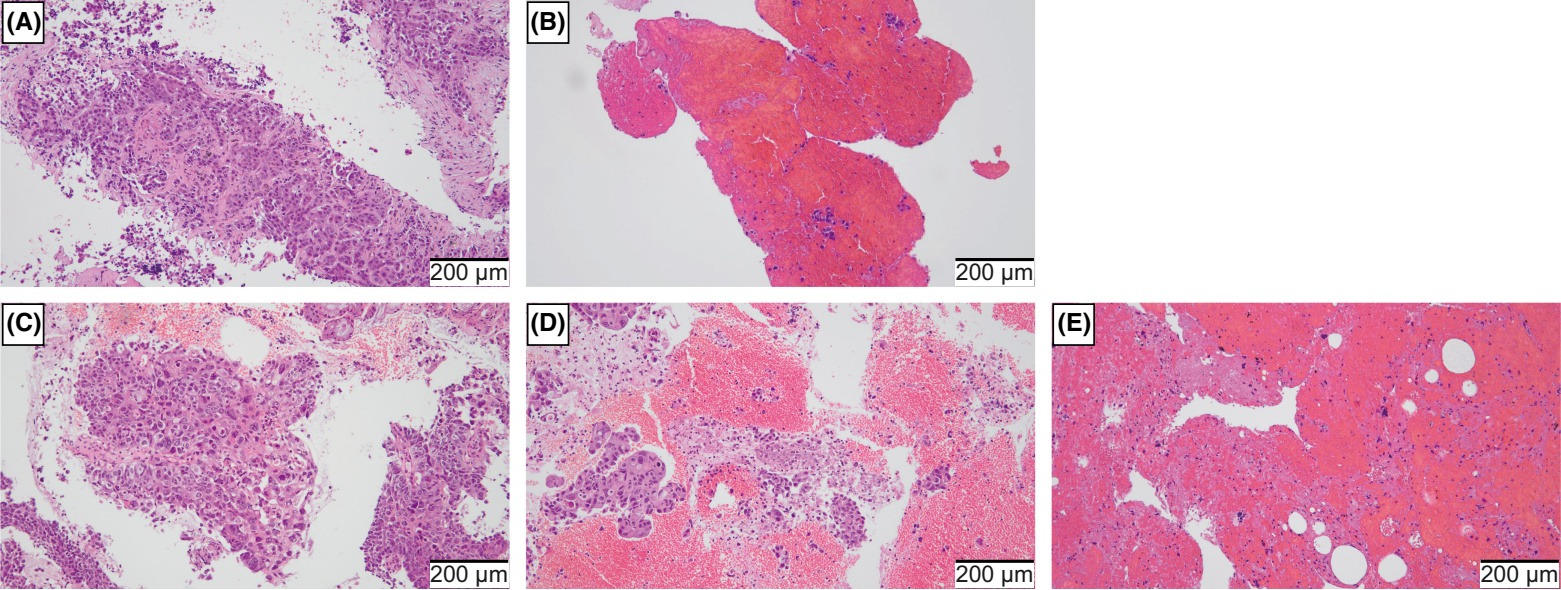
Evaluation of tissue core and blood contamination on hematoxylin–eosin‐stained slides. (A) Slide with a tissue core, (B) slide without a tissue core, (C) slide with low blood contamination, (D) slide with moderate blood contamination, and (E) slide with high blood contamination.

Specimens obtained via EBUS‐TBNA were fixed in 10% neutral buffered formalin solution for 20–28 h and embedded in paraffin (Thermo Fisher Scientific Inc.). Thin slides were prepared using formalin‐fixed paraffin‐embedded (FFPE) specimens cut to 3–4‐μm thickness with a microtome (Yamato Kohki Industrial Co., Ltd.), and histopathological examination was performed using the hematoxylin–eosin staining method. We collected data on histopathological analysis, including the presence of tissue cores, degree of blood contamination, and number of tumor cells on the slides. We defined a tissue core as a contiguous string of lung cancer tissues on a slide^25^ (Figure 1A,B). Blood contamination was classified into three levels: low (no or few blood cells affecting diagnosis), moderate (blood cells obscure a part of the specimen, but pathological diagnosis is possible), and high (many blood cells make pathological diagnosis difficult)^19^, ^26^ (Figure 1C–E). The tumor cell count was defined as the number of tumor cells on the slide.^12^ Cells that were crushed and difficult to identify as tumor cells were not counted. Experienced pathologists at Yokohama City University Medical Center performed all pathological examinations. Pathologists were blinded to the EBUS‐TBNA method used to collect the specimens.

### 2.4 NGS analysis

Patients diagnosed with NSCLC using EBUS‐TBNA with the slow‐pull or aspiration methods and who subsequently underwent NGS were selected. NGS was conducted using Oncomine Dx Target Test Multi‐CDx (ODxTT; Thermo Fisher Scientific Inc., Waltham, MA, USA) or in the clinical trial Lung Cancer Genomic Screening Project for Individualized Medicine in Asia (LC‐SCRUM‐Asia).^27^ ODxTT is used to analyze hotspot mutations using DNA derived from tumor samples and fusion genes using RNA derived from tumor samples for 46 genes (Table 1). ODxTT is approved as a companion diagnostic system in Japan that identifies changes in five driver genes: *EGFR*, *ALK*, *ROS1*, *BRAF* V600E, and *RET*.^28^ The ODxTT analysis was performed based on Ion AmpliSeq technology after submitting 5‐μm‐thick slides prepared using FFPE specimens to SRL Laboratories. We collected data on the success or failure of the DNA and RNA analyses. We defined “success” as a case in which all genetic tests for both DNA and RNA were successfully analyzed. NGS of LC‐SCRUM‐Asia was conducted on fresh‐frozen specimens collected separately. Fresh‐frozen specimens were submitted directly to NGS after collection and freezing, and therefore, the presence of tissue cores, degree of blood contamination, and tumor cell count could not be assessed under a microscope. Hence, we did not collect data on the success or failure of NGS in LC‐SCRUM‐Asia.

**TABLE 1:** Target genes that can be detected using the Oncomine Dx Target Test Multi‐CDx.

DNA							
*AKT1*	*ALK*	*AR*	*BRAF*	*CDK4*	*CTNNB1*	*DDR2*	*EGFR*
*ERBB2*	*ERBB3*	*ERBB4*	*ESR1*	*FGFR2*	*FGFR3*	*GNA11*	*GNAQ*
*HRAS*	*IDH1*	*IDH2*	*JAK1*	*JAK2*	*JAK3*	*KIT*	*KRAS*
*MAP2K1*	*MAP2K2*	*MET*	*MTOR*	*NRAS*	*PDGFRA*	*PIK3CA*	*RAF1*
*RET*	*ROS1*	*SMO*					

RNA							
*ABL1*	*ALK*	*AXL*	*BRAF*	*ERBB2*	*ERG*	*ETV1*	*ETV4*
*ETV5*	*FGFR1*	*FGFR2*	*FGFR3*	*MET*	*NTRK1*	*NTRK2*	*NTRK3*
*PDGFRA*	*PPARG*	*RAF1*	*RET*	*ROS1*			

### 2.5 Statistical analysis

We used Mann–Whitney *U*‐test to compare numerical data between the groups. We also used the chi‐square test or Fisher's exact test to compare the proportions of categorical data between the groups. Significance was determined at *p* < 0.05 with a two‐tailed *t*‐test. Statistical analyses were performed using GraphPad Prism 9 software (GraphPad Software).

## 3 RESULTS

### 3.1 Patient characteristics

**FIGURE 2:**
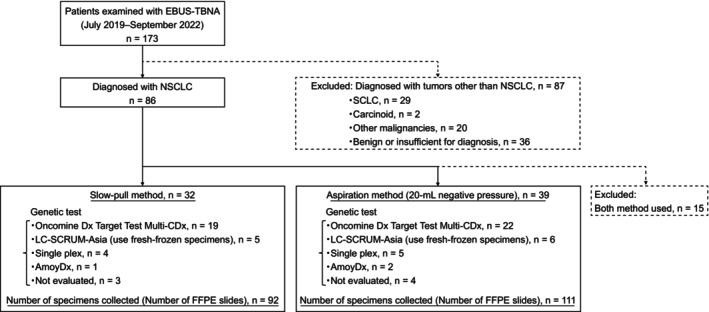
Flow diagram depicting the patient selection process. EBUS‐TBNA, endobronchial ultrasound‐guided transbronchial needle aspiration; FFPE, formalin‐fixed paraffin‐embedded; LC‐SCRUM‐Asia, Lung Cancer Genomic Screening Project for Individualized Medicine in Asia; NSCLC, non‐small cell lung cancer; SCLC, small cell lung cancer.

Figure 2 presents the patient selection flowchart. A total of 173 patients underwent EBUS‐TBNA between July 2019 and September 2022, and 86 were diagnosed with NSCLC. From these, 71 patients were included in this study after excluding 15 patients who underwent both slow‐pull and aspiration procedures. Of the included patients, 32 underwent the slow‐pull procedure alone; of these, 19 underwent ODxTT for genetic testing, and 5 submitted fresh‐frozen specimens to LC‐SCRUM‐Asia. The total number of specimens collected from the 32 patients (FFPE slides) was 92. Of the 71 patients, 39 underwent aspiration procedures; from these, 22 patients underwent ODxTT‐based genetic testing and 6 submitted fresh‐frozen specimens to LC‐SCRUM‐Asia. A total of 111 specimens (FFPE slides) were collected from 39 patients.

We observed no significant differences in patient background between the slow‐pull and aspiration method groups (Table 2). Twenty‐eight patients (87.5%) in the slow‐pull method group and 37 (94.9%) in the aspiration group underwent positron emission tomography (PET)‐computed tomography (CT) prior to EBUS‐TBNA. The patients who did not undergo PET‐CT were examined using contrast‐enhanced CT of the chest. The most common puncture sites in both groups were the subcarinal lymph nodes (13 [40.6%] in the slow‐pull method group and 18 [46.1%] in the aspiration method group) and the lower paratracheal lymph nodes (13 (40.6%) in the slow‐pull method group and 16 (41.0%) in the aspiration method group). The median maximum diameter of the puncture site was 29 (interquartile range [IQR]: 24–35) and 27 (IQR: 20–35) mm in the slow‐pull and aspiration method groups, respectively. The median short diameter of the puncture site was 19 (IQR: 14–25) and 17 (IQR: 13–31) mm in the slow‐pull and aspiration method groups, respectively. The median maximum and short diameter of the puncture sites were not significantly different between the methods (*p* = 0.31 and 0.59, respectively). The median number of specimens collected was three in both groups. The median duration of the procedure was 45 (IQR: 37–54) min for the slow‐pull method group and 39 (IQR: 33–54) min for the aspiration method group, with no significant difference (*p* = 0.29). Adenocarcinoma was the most common histology in both groups (slow‐pull method group, 17 [53.1%]; aspiration method group, 22 [56.4%]), followed by squamous cell carcinoma (slow‐pull method group, 10 [31.3%]; aspiration method group, 10 [25.6%]).

**TABLE 2:** Patient characteristics.

Characteristic	All patients (*n* = 71)	Slow‐pull method (*n* = 32)	Aspiration method (*n* = 39)	*p*
Age, years	72 (63–76)	73 (63–76)	70 (63–76)	0.48
Sex				0.44
Male	52 (73.2)	22 (68.8)	30 (76.9)	
Female	19 (26.8)	10 (31.2)	9 (23.1)	
Smoking status				0.72
Current or former	63 (88.8)	29 (90.6)	34 (87.2)	
Never	8 (11.2)	3 (9.4)	5 (12.8)	
ECOG PS				0.69
0–1	64 (90.1)	28 (87.5)	36 (92.3)	
2–3	7 (9.9)	4 (12.5)	3 (7.7)	
PET‐CT performed prior to EBUS‐TBNA	65 (91.6)	28 (87.5)	37 (94.9)	0.40
Location of target lesion				0.60
Subcarinal lymph nodes (7)	31 (43.7)	13 (40.6)	18 (46.1)	
Lower paratracheal lymph nodes (4R, 4 L)	29 (40.8)	13 (40.6)	16 (41.0)	
Interlobar and lobar lymph nodes (11R, 11 L)	6 (8.5)	2 (6.3)	4 (10.3)	
Hilar lymph nodes (10 L)	1 (1.4)	1 (3.1)	0	
Central pulmonary lesions	4 (5.6)	3 (9.4)	1 (2.6)	
Maximum diameter of the target lesion on chest CT, mm	29 (21–35)	29 (24–35)	27 (20–35)	0.31
Short diameter of the target lesion on chest CT, mm	18 (13–25)	19 (14–25)	17 (13–31)	0.59
Number of specimens collected	3 (2–3)	3 (2–4)	3 (2–3)	0.80
Duration of procedure, min	44 (34–54)	45 (37–54)	39 (33–54)	0.29
Stage				0.77
II	2 (2.8)	1 (3.1)	1 (2.6)	
III	33 (46.5)	17 (53.1)	16 (41.0)	
IV	35 (49.3)	14 (43.8)	21 (53.9)	
Postoperative recurrence	1 (1.4)	0	1 (2.5)	
Pathological diagnosis				0.87
Adenocarcinoma	39 (54.9)	17 (53.1)	22 (56.4)	
Squamous cell carcinoma	20 (28.2)	10 (31.3)	10 (25.6)	
Non‐small cell carcinoma, not otherwise specified	12 (16.9)	5 (15.6)	7 (18.0)	
PD‐L1 TPS				0.73
< 1%	23 (32.4)	10 (31.3)	13 (33.3)	
1%–49%	20 (28.2)	9 (28.1)	11 (28.2)	
≥ 50%	26 (36.6)	13 (40.6)	13 (33.3)	
Not evaluated	2 (2.8)	0	2 (5.1)	

*Note*: Data are presented as *n* (%) or median (interquartile range). The chi‐square test and Fisher's exact test were used to compare the proportions of categorical data between the groups.

Abbreviations: CT, computed tomography; ECOG PS, Eastern Cooperative Oncology Group performance status; PD‐L1 TPS, programmed death ligand 1 tumor proportion score; PET, positron emission tomography.

### 3.2 Pathological findings of FFPE slides of specimens collected via EBUS‐TBNA

**FIGURE 3:**
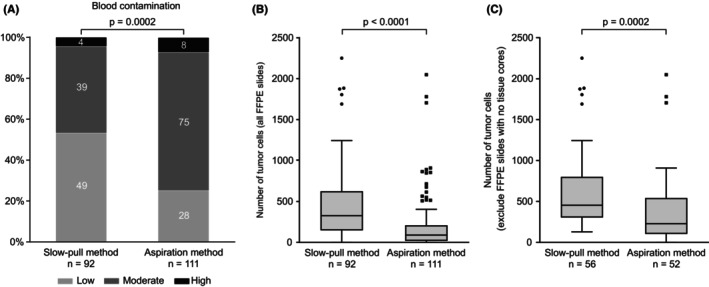
Comparison of the slow‐pull and aspiration methods with respect to the (A) extent of blood contamination, (B) number of tumor cells (all formalin‐fixed paraffin‐embedded slides), and (C) number of tumor cells (excluding formalin‐fixed paraffin‐embedded slides for which tissue cores were not collected). We used Fisher's exact test to compare the proportions of categorical data between the groups (A) and Mann–Whitney *U*‐test to compare numerical data between the groups (B, C).

The total number of specimens (FFPE slides) collected using the slow‐pull and aspiration methods was 92 and 111, respectively. The slow‐pull method group had a higher tissue core collection rate than the aspiration method group (60.9% vs. 46.9%, *p* = 0.046; Table 3). Specimens from the slow‐pull method group had less blood contamination than those from the aspiration method group (low: 53.3% vs. 25.2%; moderate: 42.4% vs. 67.6%; and high: 4.4% vs. 7.2%; *p* = 0.0002; Figure 3A). Specimens from the slow‐pull method group had a higher number of tumor cells than those from the aspiration method group (328 [IQR: 149–625] vs. 90 [IQR: 20–210], *p* < 0.0001; Figure 3B). Even after excluding FFPE slides for which tissue cores were not collected, specimens from the slow‐pull method group had a higher number of tumor cells than those from the aspiration method group (455 [IQR: 305–800] vs. 230 [IQR: 106–543], *p* = 0.0002; Figure 3C).

**TABLE 3:** Pathological findings of formalin‐fixed paraffin‐embedded slides of specimens collected via EBUS‐TBNA.

Variable	All specimens (*n* = 203)	Slow‐pull method (*n* = 92)	Aspiration method (*n* = 111)	*p*
Core tissues				0.046
Presence	108 (53.2)	56 (60.9)	52 (46.9)	
Absence	95 (46.8)	36 (39.1)	59 (53.2)	

*Note*: Data are presented as *n* (%). EBUS‐TBNA, endobronchial ultrasound‐guided transbronchial needle aspiration. The chi‐square test was used to compare the proportions of categorical data between the groups.

### 3.3 Success rate of ODxTT with FFPE slides of specimens collected via EBUS‐TBNA

FFPE slides of 41 patients (19 out of 32 in the slow‐pull method group and 22 out of 39 in the aspiration method groups) were subjected to ODxTT. DNA and RNA were successfully analyzed in 33 patients (80.5%). The slow‐pull method was superior to the aspiration method, with successful analysis of both DNA and RNA from 18 patients (94.7%) in the slow‐pull method group and 15 patients (68.2%) in the aspiration method group (*p* = 0.049; Table 4).

**TABLE 4:** Success rate of the Oncomine Dx Target Test Multi‐CDx with formalin‐fixed paraffin‐embedded slides of specimens collected via EBUS‐TBNA.

	All patients (*n* = 41)	Slow‐pull method (*n* = 19)	Aspiration method (*n* = 22)	*p*
Both DNA and RNA successfully evaluated	33 (80.5)	18 (94.7)	15 (68.2)	0.049
Only DNA failed to be evaluated	4 (9.8)	1 (5.3)	3 (13.6)	
Only RNA failed to be evaluated	2 (4.9)	0	2 (9.1)	
Both DNA and RNA failed to be evaluated	2 (4.9)	0	2 (9.1)	

*Note*: Data are presented as *n* (%). EBUS‐TBNA, endobronchial ultrasound‐guided transbronchial needle aspiration. The chi‐square test was used to compare between the groups for the percentage of both DNA and RNA that were successfully evaluated.

### 3.4 Complications of EBUS‐TBNA

Table 5 shows the complications of EBUS‐TBNA. Bacterial pneumonia occurred in one patient (3.1%) in the slow‐pull group but was resolved with antibiotic use. Bacterial pneumonia was also observed in one patient (2.6%) in the aspiration method group but was resolved with antibiotic use. In the aspiration method group, one patient (2.6%) had bloody phlegm, requiring the administration of hemostatic agents the day after EBUS‐TBNA, but it disappeared after 2 days.

**TABLE 5:** Complications of EBUS‐TBNA.

	All patients (*n* = 71)	Slow‐pull method (*n* = 32)	Aspiration method (*n* = 39)
Bacterial pneumonia	2 (2.8)	1 (3.1)	1 (2.6)
Bloody phlegm	1 (1.4)	0	1 (2.6)

*Note*: Data are presented as *n* (%). EBUS‐TBNA, endobronchial ultrasound‐guided transbronchial needle aspiration.

## 4 DISCUSSION

In this study, we demonstrated that the slow‐pull method was more effective than the aspiration method in EBUS‐TBNA for collecting specimens with a tissue core, less blood contamination, and a higher number of tumor cells. To the best of our knowledge, this is the first study to demonstrate that the slow‐pull method increases the success rate of NGS analysis in EBUS‐TBNA compared to the aspiration method.

Although the syringe aspiration method is commonly used in EBUS‐TBNA, the slow‐pull method is used as frequently as the aspiration method in EUS‐FNA of pancreatic masses.^29^ Regardless of the needle size or type, the aspiration force of the slow‐pull method is remarkably lower than that of the aspiration method (slow‐pull method, 0.6–2.9 kPa; 10‐mL negative pressure, 29.2–30.4 kPa; 20‐mL negative pressure, 45.6–46.7 kPa).^30^ This is important because blood contamination in EBUS‐TBNA specimens can hamper NGS analysis.^13^, ^19^ The weak aspiration force of the slow‐pull method is expected to suppress blood contamination in the specimens, resulting in a higher success rate of NGS.

In the present study, the slow‐pull method was found to be superior to the aspiration method in terms of tissue core collection rate, with less blood contamination. This finding is consistent with the results of a meta‐analysis of 11 studies, involving 1055 patients who underwent EUS‐FNA of pancreatin masses,^23^ indicating that the slow‐pull method is associated with a higher tissue core collection rate and less blood contamination in EBUS‐TBNA. To the best of our knowledge, only one study has compared the quality of specimens collected using the slow‐pull and aspiration methods in EBUS‐TBNA. A retrospective study involving 86 patients found that the slow‐pull method was superior to the aspiration method in terms of tissue core collection rate but not in terms of blood contamination.^19^ However, the study did not examine the number of tumor cells and the success rate of NGS analysis.

In this study, we found the slow‐pull method to be superior to the aspiration method for collecting specimens with more tumor cells. The meta‐analysis of EUS‐FNA for pancreatic masses mentioned above reported no significant difference in tumor cell counts when comparing specimens collected using the slow‐pull and aspiration methods.^23^ However, these studies focused on FFPE‐cell blocks, not FFPE‐embedded tissue, as in this study. In EBUS‐TBNA, no studies have compared tumor cell counts for specimens collected using both methods. In this study, cells that were crushed and difficult to identify as tumor cells were not counted. In specimens collected using the aspiration method, it is possible that tumor cells are crushed by the strong aspiration force and are not counted. Further extensive studies are required to verify the differences in tumor cell counts between the techniques used in this study.

The overall success rate of the ODxTT analysis was 80.5%. This is comparable to the 86.5% reported in a meta‐analysis of 21 studies, involving 1175 patients.^31^ In patients with advanced recurrent NSCLC undergoing EBUS‐TBNA, it is recommended that additional specimens be collected for genetic mutation/translocation analysis beyond the number of specimens required to confirm the diagnosis; specifically, at least three specimens should be collected.^17^ In this study, the median number of specimens collected was three for both slow‐pull and aspiration method groups. Recently, the number of specimens collected (four or more) has been reported to be an essential factor affecting the success of NGS on EBUS‐TBNA specimens.^32^ In this study, the NGS success rate may have been further improved by increasing the number of specimens collected to four or more. In recent years, the usefulness of the wet suction method, wherein the needle is filled with normal saline solution and a negative pressure is applied,^20^ and the combination of the fanning and slow‐pull methods, wherein the needle is stroked while changing its direction within the lesion,^33^ has been reported for EUS‐FNA of pancreatic masses. In EUS‐FNA of pancreatic masses, a three‐plane symmetric needle with Franseen geometry can improve the tissue core collection rate,^34^ and its effectiveness in EBUS‐TBNA is currently being assessed.^35^ Further studies are required to optimize the collection of specimens suitable for NGS in EBUS‐TBNA using various methods and puncture needles.

The results of this study did not reveal any factor that strongly recommends the use of slow‐pull method over the aspiration method when performing EBUS‐TBNA. However, as there were no significant differences in the duration of the procedure or rate of complications between the methods, it is recommended that the slow‐pull method be performed first and then switched to the aspiration method when specimens cannot be collected with the slow‐pull method.

However, this study has several limitations. First, this was a single‐center retrospective study with a relatively small number of patients. Second, the tumor content rate was not examined in this study because the tumor cells were scattered in the specimens collected using EBUS‐TBNA, making it difficult to accurately measure the tumor content rate. Third, only a portion of patients in this study underwent NGS. Despite the small number of patients included in the statistical analysis, the success rate of NGS was higher in the slow‐pull method group than in the aspiration method group. Finally, in this study, we excluded patients who underwent both procedures to compare the success rate of NGS analysis. Target lesion location and size were not significantly different between the methods, but biases such as tumor distribution within the target lesion could not be completely eliminated. Studies comparing the two methods in the same patients are necessary to avoid bias due to differences in the puncture lesions.

In conclusion, our study demonstrated that real‐world EBUS‐TBNA can be used to collect sufficient specimens suitable for NGS. The slow‐pull method in EBUS‐TBNA can quickly increase the success rate of NGS analysis without the need for additional materials. As a result, the slow‐pull method may contribute to the identification of driver gene mutations and translocations and facilitate personalized treatment of NSCLC and thus may be worth applying as a standard technique for EBUS‐TBNA. Nevertheless, prospective randomized controlled trials are needed to further understand the effectiveness of the slow‐pull method for collecting samples suitable for NGS in EBUS‐TBNA.

## AUTHOR CONTRIBUTIONS

**Yukihito Kajita:** Conceptualization (lead); data curation (lead); formal analysis (lead); investigation (lead); methodology (lead); project administration (lead); writing – original draft (lead); writing – review and editing (lead). **SHUHEI TERANISHI:** Conceptualization (lead); data curation (lead); formal analysis (lead); funding acquisition (lead); investigation (lead); methodology (lead); project administration (lead); resources (lead); software (lead); visualization (lead); writing – original draft (lead); writing – review and editing (lead). **Tomoe Sawazumi:** Conceptualization (equal); data curation (lead); formal analysis (lead); investigation (lead); methodology (lead); project administration (equal); writing – review and editing (lead). **Haruka Watanabe:** Data curation (equal); investigation (equal). **Satoshi Nagaoka:** Formal analysis (equal); investigation (equal). **Anna Tanaka:** Data curation (equal); formal analysis (equal). **Yuichirou Suzukawa:** Data curation (equal); investigation (equal). **Yuto Motobayashi:** Formal analysis (equal); investigation (equal). **Tomofumi Hirose:** Formal analysis (equal); investigation (equal). **Chihiro Maeda:** Data curation (equal); investigation (equal). **Kenichi Seki:** Formal analysis (equal); investigation (equal). **Ken Tashiro:** Formal analysis (equal); investigation (equal). **Nobuaki Kobayashi:** Writing – review and editing (equal). **Masaki Yamamoto:** Methodology (equal); writing – review and editing (equal). **Makoto Kudo:** Methodology (equal); writing – review and editing (equal). **Yoshiaki Inayama:** Supervision (lead); writing – review and editing (lead). **Takeshi Kaneko:** Supervision (lead); writing – review and editing (lead).

## FUNDING INFORMATION

The authors declare that no funds, grants, or other support were received during the preparation of this manuscript.

## CONFLICT OF INTEREST STATEMENT

The authors have no relevant financial or non‐financial interests to disclose.

## ETHICAL APPROVAL STATEMENT

This study was performed in line with the principles of the Declaration of Helsinki. Approval was granted by the Ethics Committee of Yokohama City University (Ethics approval number: F221000009).

## PATIENT CONSENT STATEMENT

This study was retrospective in nature and did not require written consent from patients. Instead, we published the study information on the hospital's website and offered patients the option to refuse participation.

## Data Availability

The data that support the findings of this study are available from the corresponding author upon reasonable request.
